# Genome-wide profiling of histone modifications in fission yeast using CUT&Tag

**DOI:** 10.1007/978-1-0716-4168-2_22

**Published:** 2025-01-01

**Authors:** Sito Torres-Garcia, Yiwen Huang, Felix Selasi Dewornu, Pin Tong, Rebecca Yeboah, Manu Shukla, Robin C. Allshire

**Affiliations:** 1 Wellcome Centre for Cell Biology and Institute of Cell Biology, School of Biological Sciences, The University of Edinburgh, Swann Building, King’s Buildings, Mayfield Road, Edinburgh EH9 3BF United Kingdom; 3 Hi-Tech Zone, Chengdu, Sichuan, China

**Keywords:** CUT&Tag, ChIP-Seq, Heterochromatin, Epigenome, Nucleosome, Histone modifications, *Schizosaccharomyces pombe*

## Abstract

Eukaryotic DNA is organised in the nucleus in the form of chromatin. Nucleosomes, the fundamental unit of chromatin, are subject to many post-translational modifications as well as compositional variations through incorporation of histone variants. These alterations play important roles in regulation of genome structure and activity. Genome-wide profiling of these regulatory features is essential to our understanding of genome function. Chromatin immunoprecipitation coupled with next generation sequencing (ChIP-Seq) is the most used method to assay genome-wide localisation in fission yeast but suffers from requirement for large amount of input chromatin, antibodies and cumbersome experimental pipeline. New methods such as Cleavage Under Targets and Tagmentation (CUT&Tag), combining the specificity of targeted cleavage and adapter insertion with the sensitivity of next-generation sequencing, enable identification and characterization of various epigenetic marks affording low input requirement as well as more streamlined protocols. However, these approaches have not been adapted for fission yeast. Here, we describe an adapted CUT&Tag protocol for epigenomic profiling in fission yeast using heterochromatic histone H3K9 methylation for benchmarking.

## 1 Introduction

Chromatin modifications and protein-DNA interactions underpin the mechanisms governing gene regulation. Chromatin immunoprecipitation followed by sequencing (ChIP-Seq) has served as a primary technique of choice for investigating protein-DNA interactions and mapping epigenetic marks across the genome (1–4). However, traditional ChIP-Seq methods require large amounts of starting material and may suffer from high background noise, limiting their applicability, particularly when studying scarce cell populations or small samples (5).

**Figure 1:**
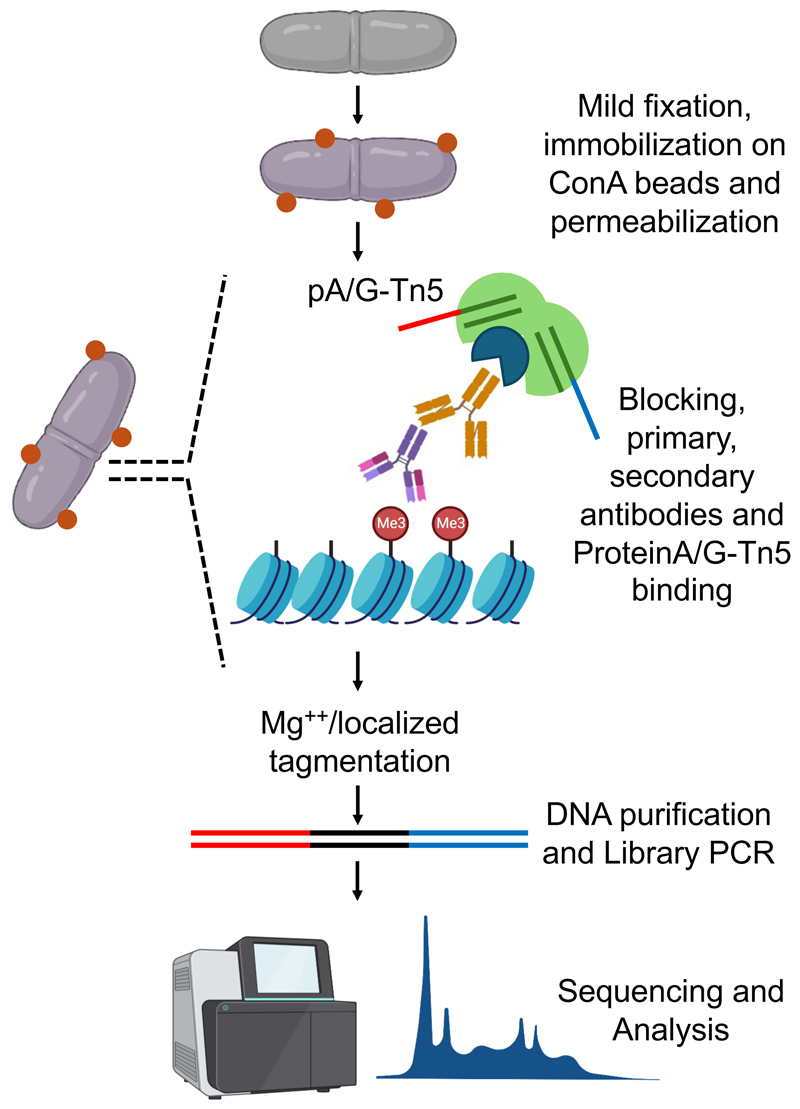
Schematic overview of CUT&Tag workflow in fission yeast. Cells are cross-linked mildly followed by spheroplasting, immobilization on ConA beads and permeabilization. ProteinA/G-Tn5 fusion protein is targeted to specific chromatin regions directed by antibodies specific to particular histone modifications or DNA binding proteins. Addition of magnesium chloride initiates localized tagmentation of chromatin. Tagmented DNA is recovered and amplified for library preparation and subsequent sequencing. “Illustration was created using Biorender.com”.

In recent years, various alternative protocols such as DamID (6), Antibody-guided chromatin tagmentation (ACT-seq) (7), ChIPmentation (8) and CUT&Run (9) have been developed to improve upon signal to noise ratio and input sample requirement for ChIP-Seq. Further, Cleavage Under Targets and Tagmentation (CUT&Tag) combines the specificity of targeted cleavage and adapter tagging enabling efficient profiling of chromatin modifications and protein-DNA interactions in small samples and single cells (10, 11). The method harnesses a fusion protein, comprising a proteinA/G-Tn5 hyperactive transposase (12), and antibodies to specific histone modifications and DNA binding proteins to localize and tag chromatin regions of interest directly in situ. Key steps in CUT&Tag methodology include permeabilization of cells or nuclei followed by incubation with antibodies to specific chromatin features. A Tn5 transposase-proteinA/G fusion is then targeted to chromatin regions marked by the user specified antibodies. A targeted tagmentation reaction results in preferential cleavage and adapter tagging of chromatin regions enriched for the protein or histone modification of interest. Subsequent amplification and sequencing of the tagged DNA fragments allows for high-resolution mapping of epigenetic features and transcription factor binding sites across the genome (Figure 1).

Here we describe an adapted stream-lined CUT&Tag protocol for genome-wide profiling of histone modifications in *Schizosaccharomyces pombe* with low number of cells as an input. Key methodological steps and reagents are described below.

## 2 Materials

### 2.1 Equipment and Reagents

1. Fission yeast growth media such as Yeast Extract with Supplements (YES) or Pombe Minimal Glutamate (PMG) (13).
Spectrophotometer or hemocytometer to estimate cell numbers in cultures.
pH meter
37% Formaldehyde solution (*see* Note 1)
2.5M Glycine
Concanavalin-A conjugated magnetic beads
2X Zymo Buffer: 40mM HEPES pH 7.6, 2M Sorbitol, 1mg/ml Zymolyase 100-T
ConA binding buffer: 20mM HEPES pH 7.6, 10mM KCl, 1mM CaCl2, 1mM MnCl2
Permeabilization buffer A (-Spermidine): 20mM HEPES pH 7.5, 150mM NaCl, 0.1% Triton X100, Roche PI (EDTA free)
  (PI added fresh, can be stored for 1 week at 4°C)
Permeabilization buffer B (+Spermidine): 20mM HEPES pH 7.5, 150mM NaCl, 0.5mM Spermidine, 0.1% Triton X100, Roche PI (EDTA free)
  (Spermidine and PI added fresh, can be stored for 1 week at 4°C)
Wash Buffer: 20mM HEPES pH 7.5, 150mM NaCl, 0.5mM Spermidine, 0.01% Tritone X100, Roche PI (EDTA free)
  (Spermidine and PI added fresh, can be stored for 1 week at 4°C)
Antibody buffer: To 2ml of wash buffer, add 0.5M EDTA to 2mM final and 15% BSA (fat-free) to 0.1% final concentration.
300 wash buffer: 20mM HEPES pH 7.5, 300mM NaCl, 0.5mM Spermidine, 0.01% Triton X100, Roche PI (EDTA free)
  (Spermidine and PI added fresh, can be stored for 1 week at 4°C)
Tagmentation buffer: 5ml 300 Wash buffer + 50 μl 1M MgCl2 (10mM final)
Rabbit anti H3K9Me3 antibody
Anti-Rabbit secondary antibody
Protein AG-Tn5, loaded
Nuclease free water
20% SDS solution
10mg/ml Proteinase K solution
Thermomixer
Bioruptor sonicator
Standard and DNA loBind microcentrifuge tubes
Phenol:Chloroform:Isoamyl alcohol pH8.0
Chloroform
Absolute Ethanol
10mM Tris pH8.0 or EB buffer
Thermocycler
Phusion High-Fidelity DNA polymerase
PCR primers for Illumina sequencing libraries
Ampure XP beads
Qubit™ dsDNA-HS Assay kit
Qubit™ Fluorometer
DynaMag™-2 Magnet
Agilent 2100 Bioanalyzer system
High Sensitivity DNA Reagents kit
High Sensitivity DNA Chips
MiniSeq High throughput Reagent Kit (150-cycles)
MiniSeq

## 3 Method

### 3.1 Fixation of cells and binding to ConA beads

1. To a 100 μl culture (10^6^ cells) add 10 μl formaldehyde from a diluted stock (1%) to achieve 0.1% final concentration). Incubate for 2.5 minutes at room temperature and then quench by addition of 5.5 μl 2.5 M glycine. Incubate at 2.5 mins at room temperature. When using higher number of cells (10^7^ cells), resuspend 10^7^ cells in 0.5 ml of the medium and add 50 μl formaldehyde and 55 μl 2.5 M glycine respectively.
2. Add 115 μl (for 10^6^ cells) or 575 μl (for 10^7^ cells) 2X-Zymo buffer containing 1mg/ml Zymolyase. Incubate at 37°C with shaking for 30-60 mins. Spheroplasting can be checked by taking a few microlitres of the cell suspension, addition of SDS to 0.1% and looking for appearance of ghosts under the microscope.
3. Prepare ConA beads during spheroplasting incubation. Use 25 μl ConA beads per sample. Invert the ConA beads a few times or mildly vortex the tube to get all the beads in suspension. Take required volume of beads (plus 25 μl for one extra sample) and put the tube on a magnet. Remove supernatant and resuspend beads in 1 ml of ConA buffer. Invert a few times, put the tube back on the magnet and remove the supernatant. Repeat this step one more time. Finally, resuspend the beads in the same volume of ConA buffer as the initial volume of the beads taken.
4. Once spheroplasting is near complete (more than 80% cells appear as ghosts), centrifuge cells at ~3800xg (~7K RPM in a small centrifuge) for 1 minute. Turn the tube around, spin again for 1 minute to get a tighter pellet and minimize cell loss. Follow this procedure at all steps where cells are collected by centrifugation.
5. Carefully take out the supernatant without disturbing the cell pellet. Attaching a 10 μl tip in front of 1 ml tip usually helps.
6. When starting with 10^7^ cells, the pellet obtained by centrigutation is large enough to be visible throughout the experimental steps. Thus, immobilization on ConA beads is not required. Skip steps 3, 7 and 8 and simply wash the cells twice in 1 ml 1X Zymo buffer (without Zymolyase 100-T) and remove supernatant.
7. Resuspend cells in 600 μl ConA binding buffer, invert tubes a few times and spin again as before. Discard supernatant completely (*see* Note 2). Repeat this step again, resulting in a total of two washes.
8. Resuspend the cell pellet in 475 μl of ConA buffer and add 25 μl washed ConA beads, rotate for 30-60 mins at room temperature. Spin briefly at 100xg to recover liquid in the lid and put tubes on magnet to collect the cells. Remove supernatant. One can also check the binding efficiency at this stage by looking for the presence of cells in the supernatant.
  Immediately proceed to Step 3.2.

### 3.2 Antibody/ProteinAG Tn-5 binding and tagmentation

1. Resuspend cells in 500 μl of permeabilization buffer, rotate/nutate at room temperature for 5 minutes. Place the tube on the magnet, remove supernatant.
2. Resuspend cells by gentle vortexing in 100 μl of freshly prepared antibody buffer (BSA, EDTA and PI added). Rotate/nutate for 30 minutes at room temperature. This step serves as a blocking step before addition of antibody.
3. Add 1 μl of the primary antibody to each sample. Rotate/nutate overnight at 4°C.
4. Briefly spin tubes at ~100xg to recover liquid in the caps. Place tubes on the magnet and remove supernatant. For 10^7^ cells, spin cells at ~3800xg (~7K RPM in the small centrifuge) as described earlier and remove supernatant carefully.
5. Mix the secondary antibody 1:100 in Wash buffer and squirt in 100 µL per sample while gently vortexing to allow the solution to dislodge the beads from the sides. For 10^7^ cells, add the diluted 100 µl secondary antibody solution to the cell pellet.
6. Place the tubes on a nutator at room temperature for 120 minutes.
7. After a quick spin, place the tubes on a magnet stand to clear and remove the liquid. For 10^7^ cells, spin cells at ~3800xg as before and remove supernatant.
8. Add 1 mL Wash buffer. Rotate for 10 min at room temperature.
  Repeat Steps 7-8 twice. Remove supernatant.
9. Mix pAG-Tn5 adapter complex in 300 Wash buffer to a final concentration of 1:20 for 50 µL per sample. (Also *see* Note 3)
10. Add 50 µL of the pAG-Tn5 mix while gently vortexing to allow the solution to dislodge most or all the beads or cells.
11. Place the tubes on a nutator at room temperature for 1 hour.
12. After a quick spin, place the tubes on a magnet stand to clear and aspirate the supernatant. For 10^7^ cells, spin cells at ~3800xg as before and remove supernatant.
13. Add 1 mL 300 Wash buffer. Invert 10x or gently vortex to allow the solution to dislodge most or all the beads or cells.
14. Collect beads on the magnet or cells by centrifugation. Repeat steps 12-13 twice.
15. After a quick spin, place the tube on the magnet stand to clear and remove the liquid. For 10^7^ cells, spin cells at ~3800xg as before and remove the supernatant.
16. Add 300 µL Tagmentation buffer while gently vortexing.
17. Incubate at 37 ºC for 1 hour in a water bath or incubator.
18. To stop tagmentation and solubilize DNA fragments, add 10 µL 0.5M EDTA, 3 µL 10% SDS and 5 µL 10 mg/mL Proteinase K to each sample.
19. Incubate overnight at 55 ºC to digest proteins and reverse cross-links.

### 3.3 Recovery of tagmented DNA

1. Put tubes on a magnet to separate the beads and take supernatant. Skip this step when starting with higher number of cells and no ConA bead immobilization was performed.
2. Sonicate on Bioruptor for 1 cycle of 30 seconds (high setting) avoiding extensive DNA fragmentation. This should be enough to break cells which are already weakened by zymolyase treatment and allow for better recovery of DNA at later steps.
3. Add 300 µl of phenol:chloroform:isoamyl alcohol (pH 8.0) to each sample, vortex full speed for 2-3 seconds and spin for 5 minutes, room temperature, at 16,000xg.
4. Collect the aqueous phase, add 300 μL Chloroform and invert ~10x to mix (do not vortex). Centrifuge 5 minutes, room temperature, at 16,000xg.
5. Remove the aqueous layer by pipetting to a fresh 1.5 mL tube containing 750 µL 100% ethanol, pipetting up and down to mix (inversion is also fine, make sure to mix well). No carrier (e.g. glycogen) is needed due to presence of high amounts of endogenous RNA and DNA relative to tagmented fragments.
6. Chill on ice and centrifuge for at least 10 minutes at 4°C 16,000xg.
7. Carefully remove the supernatant and dab on a paper towel. A visible pellet is typically not seen.
8. Rinse in 1 mL 100% ethanol and centrifuge 1 minute at 4°C 16,000xg.
9. Carefully pour off the supernatant and drain on a paper towel. Air dry.
10. When the tube is dry, resuspend the DNA in 25-30 μL EB and vortex on full to dissolve.

### 3.4 PCR amplification of libraries

**Figure 2:**
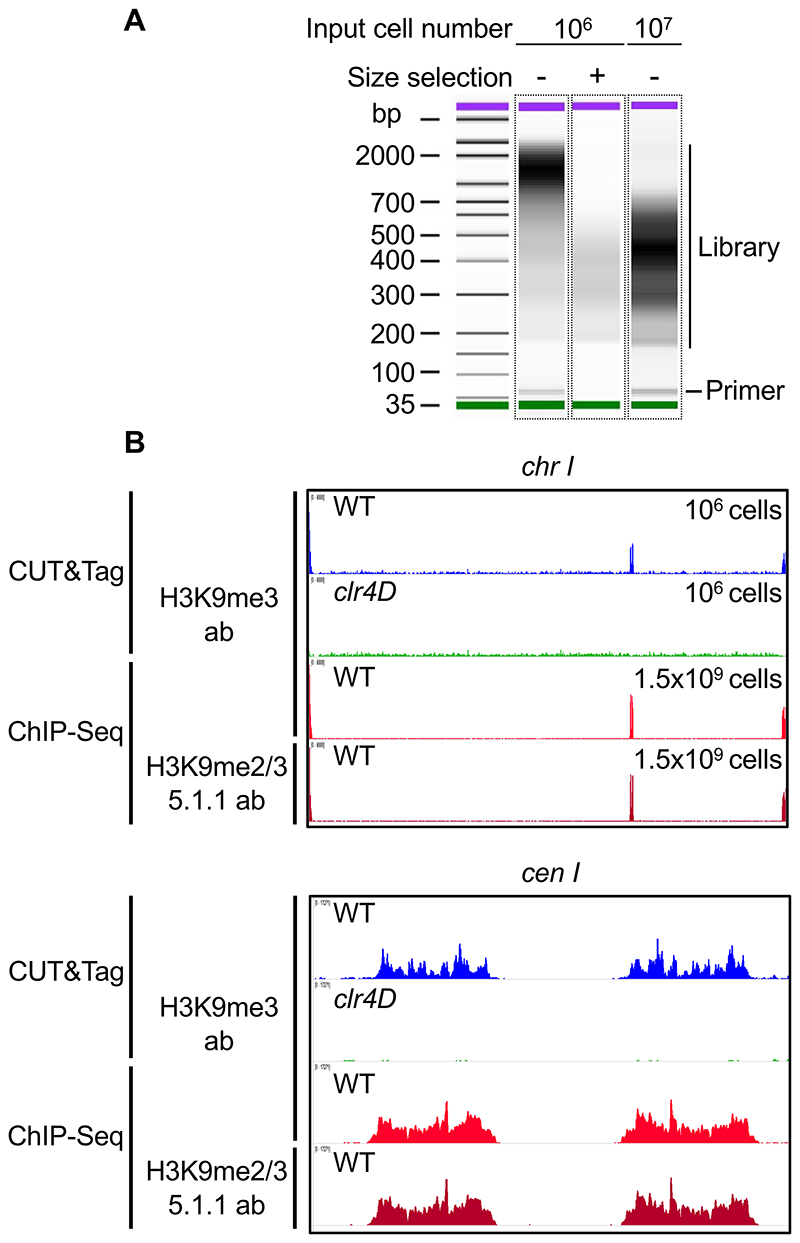
Effective detection of H3K9 methylation by CUT&Tag in fission yeast cells. A. Typical BioAnalyzer traces obtained from representative CUT&Tag experiments in *S. pombe*. Input cell numbers, library fragment distributions and presence of primers are indicate. B. Representative profiles generated by CUT&Tag for H3K9 methylation are compared to typical profiles obtained through standard ChIP-Seq. H3K9me2/3 distribution across chromosome I is shown in the upper pannel, antibodies used and input cell numbers for respective datasets are indicated. *clr4D* represents a negative control where the sole H3K9 methyltransferase gene *clr4*^*+*^ is deleted resulting in no heterochromatin present (shown in green). A zoomed view of *centromere 1* region is shown in the lower panel for indicated datasets.

1. Set up the following PCR reaction on ice in a thin-wall 0.5 ml PCR tube, using a different barcode for each sample. Use primers as described in (14). Save remaining DNA as a backup.
  15 μl adapter-ligated DNA
  10 μl 5x Phusion HF buffer
  1 μl 10 mM dNTPs
  2.5 μl 10 µM Universal or barcoded i5 primer
  2.5 μl 10 µM barcoded i7 primer
  1.5 μl DMSO
  0.5 μl Phusion polymerase
  17 μl ddH2O
2. Mix and quick spin. Place in thermocycler and begin cycling program with heated lid (*see* Notes 4 and 5).
  Cycle 1: 58°C for 5 minutes (gap filling step)
  Cycle 2: 72°C for 5 minutes (gap filling step)
  Cycle 3: 98°C for 30 seconds
  Cycle 4: 98°C for 10 seconds
  Cycle 5: 65°C for 10 seconds
  Cycle 6: 72°C for 10 seconds
  Repeat Cycles 4-6 12-16 times.
  72°C for 5 min and hold at 10°C
3. Do a 1:1.6 X Ampure XP bead cleanup (80 μl beads). Finally elute in 25-30 μl EB.
4. Check 1 μl library sample on Bioanalyzer using High Sensitivity DNA kit to assess library fragment size distribution and presence of adapters. See Figure 2A for typical library profiles (also *see* Notes 6 and 7). If free primers are present in significant amounts, an additional 1:1.3 X Ampure XP bead clean up could be performed to remove the primer content.
5. Measure the library concentration on Qubit™ fluorometer using the Qubit™ dsDNA-HS Assay kit using 2-5 μl of purified libraries. Use the library average size information obtained from Bioanalyzer profiles in combination with library concentrations calculated from Qubit™ fluorometer for multiplexing and creating the final library pool for sequencing.
6. Libraries can now be sequenced on all Illumina sequencers. Typically, two to five million reads provide sufficient coverage for fission yeast CUT&Tag libraries.

### 3.5 Data analysis

Approximately 15 million 75-bp paired-end reads were produced for each sample.

1. Raw reads are de-multiplexed and trimmed using Cutadapt (v4.4) (15) to remove adaptor contamination.
2. Trimmed reads arew aligned to the *S. pombe* reference genome (*972h*^–^, ASM294v2.20) using Bowtie 2 (v2.3.3) (16).
3. Resulting bam files are processed using Samtools (v1.3.1) (17) for sorting and indexing. Coverage bigwig files were generated by BamCoverage (deepTools v2.0) (18).
4. The complete Snakemake (19) workflow used for data analysis is available at https://github.com/SitoTorres/CUT-Tag_S.pombe_Snakemake.

## Notes

1. Formaldehyde is toxic upon skin exposure, inhalation or ingestion. Use appropriate safety gear such as a lab coat, eye protection and gloves. Use you institution specific containment and disposal routes for all the steps involving formaldehyde containing solutions.
2. We have observed that presence of sorbitol inhibits the Zymolyase 100-T digested spheroplasts from binding to ConA beads. Care should be taken to remove all the supernatant from steps 3.1.5 and 3.1.7 to ensure sorbitol is efficiently removed.
3. ProteinA-Tn5 could also be used instead of ProteinAG-Tn5. Antibody host species should be taken in consideration when utilizing ProteinA-Tn5. Additionally, adapter loaded ProteinA-Tn5 could be produced in house as described (11).
4. Alternative polymerases could be used for library amplification. The initial PCR cycles at 58°C and 72°C serve to fill the gaps left behind by tagmentation reaction. Thus, care should be taken when using a Hot Start DNA polymerase and PCR reactions should be heated to activate the DNA polymerase before addition of tagmented template DNA.
5. Do not add extra PCR cycles to see a signal by Bioanalyser (or Tapestation). If no nucleosomal DNA ladder like pattern is observed in library electrophoreses, try increasing the starting number of cells and switch to the alternative protocol without ConA bead immobilisation. Extra PCR cycles would reduce the complexity of the library and may result in very high level of PCR duplicates.
6. It is recommended that preferential tagmentation in the expected target region versus a negative control locus should be checked by performing qPCR on DNA obtained from step 3.3.10 or amplified library from step 3.4.4. This could be done designing primers for target amplicons limited to 80-100 base pairs. Alternatively, one of the qPCR primers could be designed to target the adapter region while the other primer is designed to target the genomic region of interest.
7. High molecular weight DNA (between 700-2000 bp) fragments are often dominant in CUT&Tag libraries when initial cell number is low (10^6^ cells) and ConA bead immobilization method is utilized. Additional right-hand selection using Ampure XP beads could be performed to remove larger fragments (see (20) for more details).
